# Dynamic changes and early predictive value of branched-chain amino acids in gestational diabetes mellitus during pregnancy

**DOI:** 10.3389/fendo.2022.1000296

**Published:** 2022-10-14

**Authors:** Xiaoxin Wang, Ya Zhang, Wei Zheng, Jia Wang, Yuanyuan Wang, Wei Song, Shengnan Liang, Cuimei Guo, Xu Ma, Guanghui Li

**Affiliations:** ^1^ Department of Obstetrics, Beijing Obstetrics and Gynecology Hospital, Capital Medical University, Beijing Maternal and Child Health Care Hospital, Beijing, China; ^2^ Department of Genetics, National Research Institute for Family Planning, Beijing, China; ^3^ Environmental and Spatial Epidemiology Research Center, National Human Genetic Resources Center, Beijing, China

**Keywords:** branched-chain amino acids, gestational diabetes mellitus, first trimester of pregnancy, prediction, biomarkers

## Abstract

**Objective:**

Branched-chain amino acids (BCAAs) are closely associated with type 2 diabetes mellitus, but their roles in gestational diabetes mellitus (GDM) are still controversial. This study aims to explore the dynamic changes of BCAAs during pregnancy and identify potential early biomarkers for GDM.

**Methods:**

This study is a nested case-control study involved 49 women with GDM and 50 age- and body mass index (BMI)-matched healthy pregnant women. The dynamic changes of valine (Val), isoleucine (Ile), and leucine (Leu) were detected in the first (8-12 weeks) and second trimesters (24-28 weeks) by liquid chromatography-mass spectrometry.

**Results:**

Serum Val, Ile, and Leu were higher in GDM patients than in controls in the first trimester. Compared with the first trimester, the serum Val, Ile, and Leu in GDM patients were decreased in the second trimester. In addition, Val, Ile, and Leu in the first trimester were the risk factors for GDM, and Ile presented a high predictive value for GDM. Ile + age (≥ 35) + BMI (≥ 24) exhibited the highest predictive value for GDM (AUC = 0.902, sensitivity = 93.9%, specificity = 80%).

**Conclusion:**

Maternal serum Ile in the first trimester was a valuable biomarker for GDM. Ile combined with advanced maternal age and overweight may be used for the early prediction of GDM.

## Introduction

Gestational diabetes mellitus (GDM) is a condition of glucose intolerance that occurs during pregnancy (1). The incidence of GDM is increasing globally, which accounts for about 1 to 14% in different countries depending on the ethnicity and social economy (2). Ageing, obesity, and a sedentary lifestyle are the main reasons for the high morbidity of GDM (3). GDM contributes to the occurrence of diverse adverse pregnancy outcomes, such as cesarean section, macrosomia, hyperbilirubinemia, hypoglycaemia, respiratory distress syndrome, and even stillbirth (4). GDM can also increase the risk of type 2 diabetes mellitus (T2DM), metabolic syndrome, and cardiovascular diseases in pregnant women (5–7). In clinical practice, GDM is usually diagnosed until the second trimester (24-28 weeks). Early identification of high-risk individuals is helpful to take preventive strategies to reduce the risk of GDM and adverse perinatal outcomes (8). Therefore, identifying of biomarkers for early detection of GDM is urgently needed.

Branched-chain amino acids (BCAAs), including valine (Val), isoleucine (Ile), and leucine (Leu) are essential amino acids that play important roles in energy homeostasis, nutrition metabolism, and immunity (9). Growing evidence has shown that BCAAs are closely associated with the risk of T2DM. A pooled cohort analysis has shown that long-term dietary intake of Leu, Ile, and Val contributes to the occurrence of T2DM (10). A metabolomic study has indicated that plasma BCAAs is a predictive marker of T2DM (11). A meta-analysis based on 27 cross-sectional and 19 prospective publications has shown that the abnormal increase of Leu, Ile, and Val elevates the risk of T2DM by about 35% (12). Similarly, it has also been reported that the increased plasma levels of BCAAs are positively correlated with an increased risk of progression from GDM to T2D later in life (13). The above studies confirm that the plasma BCAAs are predictors of T2DM.

The pathogenesis of GDM is similar to that of T2DM, but the roles of BCAAs on GDM are still controversial. Evidence has determined that the serum levels of Ile and Leu in the first trimester are increased in GDM patients than healthy pregnant women, which may predict GDM (14, 15). However, Lewis et al. (2015) have shown that the serum BCAAs in the first trimester are not significantly changed in pregnant women with GDM (16). Some previous studies have indicated that the BCAAs in the second trimester are predictors of GDM. A meta-analysis involving 432 subjects showed that the plasma concentrations of BCAAs are higher in the GDM group than those in the control group (17). Park S, et al. (2015) have found that the circulating concentration of Val at 24-28 weeks of pregnancy is independently and positively associated with GDM (18). On the contrary, Rahimi N, et al. (2017) have found that asparagine, threonine, aspartic acid, phenylalanine, glutamine, and arginine are risk factors of GDM in women with gestational age ≥ 25 weeks, while Leu, Ile, and Val are not (19). In addition, the differences of BCAAs between the first and second trimesters are rarely reported. Zhao et al. (2019) have found that the serum Leu/Ile is decreased in the second trimester (26.47 ± 2.28 weeks) than the first trimester (12.97 ± 1.00 weeks) in GDM patients, but is not changed in the controls (20). Since the roles of BCAAs in GDM are still controversial, it is urgent to reveal the dynamic changes of BCAAs in different trimesters and reveal the potential predictive value for GDM.

In this study, the serum levels of BCAAs in patients with GDM were dynamically monitored in the first and second trimesters, to reveal the correlations between BCAAs and GDM in different trimesters. Furthermore, we explored whether BCAAs could be used as biomarkers for early detection of GDM, and this may provide clinical guidance for pregnant women with a risk of GDM.

## Materials and methods

### Participants

The participants in this nested case-control study were enrolled from Beijing Obstetrics and Gynecology Hospital, Capital Medical University (Third-grade class-A specialized hospital, Beijing, China). Pregnant women at 18-44 years old with a singleton pregnancy and complete clinical information were included, and those with pre-existing GDM/T2DM, hypertension, nephropathy, and/or cardiovascular diseases were excluded. Subjects who diagnosed with diabetes or impaired glucose tolerance in the first trimester (8-12 weeks) were also excluded. GDM was diagnosed in the second trimester (24-28 weeks) according to American Diabetes Association (ADA) criteria *via* a 75g OGTT. The diagnostic criteria of OGTT for GDM were fasting blood glucose (FGB) ≥ 5.1 mmol/L,1 h blood glucose ≥ 10.0 mmol/L, and/or 2 h blood glucose ≥ 8.5 mmol/L (21). A total of 49 women diagnosed with GDM were finally enrolled. Correspondingly, 50 normoglycaemic women were matched for age (± 3 years) and pre-pregnancy BMI to GDM women in the same cohort (Control group). After the definite diagnosis of GDM, these patients received diet and exercise guidance, and 3 of them had received insulin injection. The current study was approved by the Ethics Committee of Beijing Obstetrics and Gynecology Hospital (2017-KY-015-01) following the Declaration of Helsinki. Written informed consents were obtained from all cases.

### Collection of clinical data

The body weight and height of enrolled participants were routinely measured by two experienced doctors every month. The pre-pregnancy body weight is self-reported by the participants. Overweight was defined as BMI ≥ 24 in accordance with the “Guidelines for prevention and control of overweight and obesity in Chinese adults (2006) (22)”. Other relevant clinical data, such as age, pregnancy and delivery history, disease history, etc. were collected from medical records.

### Detection of fasting plasma glucose and serum lipids and BCAAs

After overnight fasting, venous blood samples were collected from participants in the first and second trimesters. The FPG and serum lipids, including cholesterol (CHOL), triglyceride (TG), high-density lipoprotein (HDL), and low-density lipoprotein (LDL) were directly detected by an automatic biochemical analyzer (CI16200, Abbott, Abbott Park, IL, USA) in the first trimester. The serum levels of BCAAs (Val, Ile, and Leu) were detected by liquid chromatography-mass spectrometry on an Agilent 1260 series HPLC system (Agilent Technologies, Palo Alto, CA, USA) coupled to a QTRAP^®^ 4500 mass spectrometer (AB SCIEX, Foster City, CA, USA) in both the first and second trimesters. The mobile phase was 80% acetonitrile (acetonitrile/water) containing 0.1% formic acid. The detail settings are as follows: speed of the pump, 140 μL/min × 0.2 min to 30 μL/min × 1.0 min to 300 μL/min × 0.2 min; ESI source, positive mode; curtain gas, 14 psi; nebulizer gas, 40 psi; auxiliary gas 45 psi; ion spray voltage, 5500 V; source temperature, 580°C; declustering potential energy, 35 V; collision energy, 30 eV. BCAAs were measured by a neutral loss scan of 102 Da (scan range 125–340 Da) and multiple reaction monitoring. Applied Biosystems Analyst software (version 1.6) was used to control the system and to process the data.

### Statistical analyses

The sample size was calculated by 2-Sample, 2-Sided Equality. Ile was used as the primary variable based on a previous study by Jiang et al. (2020) (15). A minimum sample size of 21 participants was needed in each group for 90% power and 95% confidence interval (α = 0.05). Statistical analyses were performed by SPSS 20.0 (SPSS Inc., Chicago, IL, USA). Continuous variables met normal distribution were expressed as mean ± standard deviation (SD), and comparison between two groups was analyzed by Student’s t-test. Categorical variables were expressed as number (N), and comparison between two groups was determined by X^2^ test. Although the enrolled women in the control group were matched for BMI, those in the GDM group still exhibited a higher pre-pregnancy BMI. Therefore, the parameters with significantly difference between the GDM and control groups (including pre-pregnancy BMI) were adjusted for analyzing the correlations of serum BCAAs (in first and second trimesters) with GDM in separate logistic regression models. In addition, receiver operating characteristic (ROC) curves were established according to the exact diagnosis of GDM and probability of GDM predicted by BCAAs. Advanced age (≥ 35) + overweight (BMI ≥ 24) were combined with BCAAs to improve the recognition ability of ROC curves. AUC, sensitivity, and specificity that calculated from ROC curves were used to evaluate the predictive value of BCAAs for GDM in the first trimester.

## Results

### The clinical characteristics of patients with GDM

A total of 49 pregnant women with GDM and 50 normal controls were enrolled in this study. The clinical characteristics of the study population were presented in **Table 1**. No significant differences were observed on the age, height, pre-pregnancy weight, birth weight of pregnant women, gravida, parity, family history of DM and hypertension, history of smoking and drinking, and neonatal sex between patients with GDM and controls. Notably, the pre-pregnancy BMI is significantly higher in patients with GDM than that in controls (P = 0.003). GDM is more prevalent in pregnant women with assisted reproduction technology (P = 0.042) and poor pregnancy history (including spontaneous abortion, birth defects, stillborn fetus, and early neonatal death, P = 0.025). In addition, significantly shorter gestational week of delivery and higher neonatal weight are revealed in patients with GDM than those in the controls (P = 0.019 and 0.048, respectively). Adverse perinatal outcomes are only observed in GDM patients, including 1 pre-eclampsia and 1 low birth weight (**Table 1**).

**Table 1:** The clinical characteristics of patients with gestational diabetes mellitus (GDM) and controls.

Parameters	Control (N = 50)	GDM (N = 49)	t/x^2^	P value
Age (years)	32.00 ± 3.02 (27-40)	33.37 ± 3.97 (26-42)	-1.933	0.056
Height (cm)	163.63 ± 5.76	161.88 ± 5.02	1.613	0.110
Pre-pregnancy weight (kg)	55.81 ± 8.29	58.68 ± 7.87	-1.768	0.080
Pre-pregnancy BMI	20.79 ± 2.52	22.38 ± 2.71	-3.014	0.003^**^
Birth weight of pregnant women (N)			0.24	0.889
2.5-4 kg	45	43		
> 4kg	3	3		
< 2.5 kg	2	3		
Gravida (N)			1.74	0.418
1	26	21		
2	16	15		
≥ 3	8	13		
Parity (N)			0.08	0.776
Primiparous	34	32		
≥ 2	16	17		
Assisted reproduction technology	2	8^a^	4.14	0.042^*^
Family history of DM (N)	6	11	1.90	0.168
Family history of hypertension (N)	12	13^c^	0.23	0.635
Poor pregnancy history (N)	2^d^	9^ae^	5.01	0.025^*^
Smoking (N)	1	2^c^	0.44	0.509
Drinking (N)	4	5^c^	0.23	0.630
Gestational week of delivery	39.08 ± 0.97	38.62 ± 0.95^b^	2.384	0.019^*^
Neonatal weight (g)	3254.60 ± 326.90	3391.60 ± 345.46^b^	-2.007	0.048^*^
Neonatal sex		^b^	0.50	0.479
Male	23	25		
Female	27	22		
Adverse perinatal outcomes	0	2^f^	2.08	0.149

BMI, Body Mass Index; DM, Diabetes mellitus; Val, valine; Ile, isoleucine; Leu, leucine. a, b, and c represented the data of 1, 2, and 3 cases were missing, respectively; d included 1 spontaneous abortion and 1 birth defects; e included 6 spontaneous abortion, 2 stillborn fetus, and 1 early neonatal death. f included 1 pre-eclampsia and 1 low birth weight. ^*^P < 0.05, ^**^P < 0.01, Control vs. GDM.

### The plasma glucose and serum lipids in patients with GDM

In the first trimester, the fasting plasma glucose (FPG), and serum levels of CHOL, TG, and LDL are significantly higher in patients with GDM than those in the controls (P < 0.05). No significantly different is revealed on serum HDL between GDM and control groups. In addition, OGTT in the second trimester shows significantly higher plasma glucose in patients with GDM at different time points compared with those in controls (P < 0.001) (**Table 2**).

**Table 2:** The serum lipids, fasting plasma glucose (FPG), and oral glucose tolerance test (OGTT) in patients with gestational diabetes mellitus (GDM) and controls.

Parameters	Control (N = 50)	GDM (N = 49)	t	P value
Serum lipids in the first trimester (8-12 weeks)				
CHOL	4.11 ± 0.65	4.46 ± 0.73	-2.566	0.012^*^
TG	1.10 ± 0.41	1.36 ± 0.61	-2.468	0.015^*^
HDL	1.48 ± 0.29	1.48 ± 0.30	0.048	0.962
LDL	2.07 ± 0.49	2.34 ± 0.60	-2.504	0.014^*^
FPG in the first trimester (8-12 weeks)	4.65 ± 0.37	4.86 ± 0.49	-2.366	0.020^*^
OGTT in the second trimester (24-28 weeks)				
0 h (Fasting)	4.46 ± 0.32	4.85 ± 0.49	-4.641	< 0.001^***^
1 h	7.25 ± 1.49	9.80 ± 1.53	-8.372	< 0.001^**^
2 h	6.28 ± 1.14	8.45 ± 1.31	-8.827	< 0.001^***^

CHOL, cholesterol; TG, triglyceride; HDL, HDL, high-density lipoprotein; LDL, low-density lipoprotein. ^*^P < 0.05, ^**^P < 0.01, ^***^P < 0.001, Control vs. GDM.

### The dynamic changes of BCAAs in the first and second trimesters

**Figure 1:**
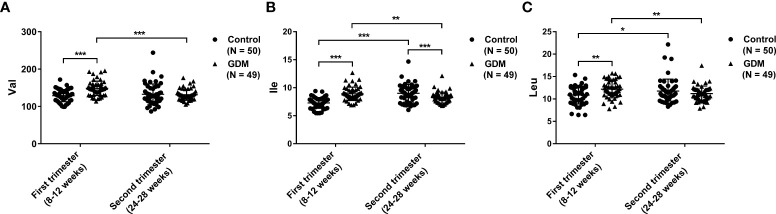
The dynamic changes of serum branched-chain amino acids (BCAAs) in patients with gestational diabetes mellitus (GDM) and normal controls in the first and second trimesters. **(A)** Valine (Val); **(B)** Isoleucine (Ile); **(C)** Leucine (Leu). The differences on the mean levels of BCAAs between GDM (N = 49) and control (N = 50) groups for different trimesters are tested using independent Student’s t-test in the first or second trimester, respectively. The differences on the mean levels of BCAAs between the first and second trimesters for different groups are tested using paired Student’s t-test in the same cohort of GDM (N = 49) or control (N = 50) groups, respectively. ^*^P < 0.05, ^**^P < 0.01, ^***^P < 0.001.

The dynamic serum changes of BCAAs were measured in the first and second trimesters. In the first trimester, the serum levels of Val, Ile, and Leu are significantly higher in patients with GDM than those in the controls (P < 0.01). However, the serum level of Ile in the second trimester is significantly lower in patients with GDM than that in controls (P < 0.01). There no significantly differences on the serum Val and Leu are revealed between GDM patients and controls in the second trimester. For pregnant women with GDM, the serum levels of Val, Ile, and Leu are significantly decreased in the second trimester compared with those in the first trimester (P < 0.01). On the contrary, the serum levels of Ile and Leu in the controls are significantly increased in the second trimester compared with those in the first trimester (P < 0.05). No significant difference is observed on Val level between the first and second trimesters in the controls (**Figures 1A–C**, and **Table 3**).

**Table 3:** The serum levels of branched-chain amino acids (BCAAs) in patients with gestational diabetes mellitus (GDM) and controls.

Parameters	First trimester (8-12 weeks)		t	P value	r value	Second trimester (24-28 weeks)		t	P value	r value
	Control (N = 50)	GDM (N = 49)				Control (N = 50)	GDM (N = 49)			
Val	128.94 ± 16.33	148.52 ± 19.34	-5.448	< 0.001^***^	0.450	132.56 ± 27.92	131.34 ± 14.89^###^	0.270	0.788	0.027
Ile	7.28 ± 0.96	8.92 ± 1.19	-7.509	< 0.001^***^	0.604	9.01 ± 1.58^###^	8.25 ± 0.97^##^	2.882	0.005^**^	0.278
Leu	10.86 ± 1.99	12.17 ± 1.92	-3.328	0.001^**^	0.318	11.76 ± 2.67^#^	11.20 ± 1.68^##^	1.257	0.212	0.125

Val, valine; Ile, isoleucine; Leu, leucine. r value represented effect size. ^**^P < 0.01, ^***^P < 0.001, Control vs. GDM; ^#^P < 0.05, ^##^P < 0.01, ^###^P < 0.001, First trimester vs. Second trimester.

### Serum BCAAs in the first trimester are the risk factors of GDM

Since there are significant changes in serum BCAAs in GDM, the potential risk factors of GDM are subsequently determined by logistic regression analysis. After adjusted for age, pre-pregnancy BMI, and weight gain in the first trimester (model 1), the Val, Ile, and Leu in the first trimester are determined as the risk factors of GDM (P < 0.01). In a similar model adjusted for age, pre-pregnancy BMI, and weight gains in both the first and second trimesters (model 2), only the Ile in the second trimester is detected to be the risk factor of GDM (P < 0.01). Based on the model 1 and 2, serum lipids and FPG in the first trimester are combined for adjusting in the regression model 3 and 4. Some clinical parameters that are significantly different between the GDM and control groups were further enrolled in the regression model 5 and 6, including assisted reproduction technology, poor pregnancy history, gestational week of delivery, and neonatal weight. In these models, the Val, Ile, and Leu in the first trimester, and the Ile in the second trimester are consistently determined as the risk factors of GDM (**Table 4**).

**Table 4:** Logistic regression analysis of the BCAAs in the first and second trimesters associated with gestational diabetes mellitus (GDM).

Parameters	First trimester (8-12 weeks)			Second trimester (24-28 weeks)		
	B	P value	OR (95%CI)	B	P value	OR (95%CI)
	Model 1^a^			Model 2^b^		
Val	0.062	< 0.001^***^	1.064 (1.032-1.098)	-0.008	0.468	0.992 (0.971-1.014)
Ile	1.559	< 0.001^***^	4.752 (2.420-9.330)	-0.673	0.004^**^	0.510 (0.324-0.804)
Leu	0.354	0.005^**^	1.425 (1.115-1.823)	-0.203	0.112	0.817 (0.636-1.048)
	Model 3^c^			Model 4^d^		
Val	0.073	< 0.001^***^	1.076 (1.039-1.115)	-0.007	0.596	0.993 (0.969-1.018)
Ile	1.795	< 0.001^***^	6.017 (2.748-13.174)	-0.762	0.003^**^	0.467 (0.283-0.770)
Leu	0.395	0.003^**^	1.485 (1.144-1.927)	-0.214	0.114	0.807 (0.619-1.053)
	Model 5^e^			Model 6^f^		
Val	0.079	< 0.001^***^	1.082 (1.043-1.123)	-0.10	0.464	0.990 (0.965-1.016)
Ile	1.993	< 0.001^***^	7.336 (2.903-18.535)	-0.924	0.002^**^	0.397 (0.223-0.707)
Leu	0.440	0.003^**^	1.552 (1.167-2.065)	-0.272	0.075	0.762 (0.565-1.028)

Val, valine; Ile, isoleucine; Leu, leucine; OR, odd ratio; CI, confidence interval. B value presents regression coefficient. ^a^ represented the parameters were adjusted for age, pre-pregnancy BMI, and weight gain in the first trimester; ^b^ represented the parameters were adjusted for age, pre-pregnancy BMI, and weight gains in the first and second trimesters; ^c^ represented the parameters were adjusted for ^a^ combined with serum lipids (cholesterol, triglyceride, high-density lipoprotein, and low-density lipoprotein) and fasting plasma glucose in the first trimester; ^d^ represented the parameters were adjusted for ^b^ combined with serum lipids and fasting plasma glucose in the first trimester; ^e^ represented the parameters were adjusted for ^c^ combined with assisted reproduction technology, poor pregnancy history, gestational week of delivery, and neonatal weight; ^f^ represented the parameters were adjusted for ^d^ combined with assisted reproduction technology, poor pregnancy history, gestational week of delivery, and neonatal weight. ^**^P < 0.01, ^***^P < 0.001.

### The value of increased BCAAs in the first trimester for the prediction of GDM

**Figure 2:**
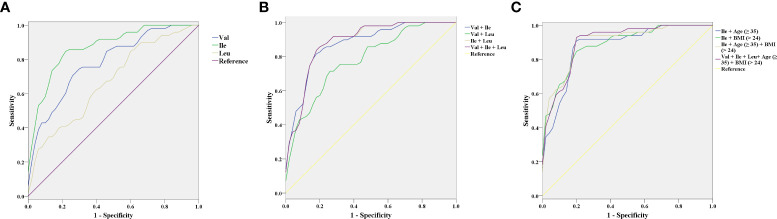
The receiver operating characteristic (ROC) curves of branched-chain amino acids (BCAAs) in the first trimester for the prediction of gestational diabetes mellitus (GDM). **(A)** Valine (Val), Isoleucine (Ile), and Leucine (Leu). **(B)** Val + Ile, Val + Leu, Ile + Leu, and Val + Ile + Leu. **(C)** Ile + age (≥ 35), Ile + BMI (≥ 24), Ile + Age (≥ 35) + BMI (≥ 24), and Val + Ile + Leu+ Age (≥ 35) + BMI (≥ 24).

We evaluated the predictive value of BCAAs in the first trimester for GDM. When single BCAA is independently used, Ile (AUC = 0.872, sensitivity = 85.7%, specificity = 78%) exhibits the highest predictive value for GDM, followed by Val (AUC = 0.782, sensitivity = 75.5%, specificity = 70%). The predictive value of Leu (AUC = 0.669, sensitivity = 89.8%, specificity = 36%) for GDM is greatly limited by low specificity (**Figure 2A** and **Table 5**). Next, the predictive value of BCAAs combinations for GDM is analyzed. Comparing with Ile with an independent high predictive value, the predictive value of Val + Leu (AUC = 0.781, sensitivity = 67.3%, specificity = 78%) is relatively lower. Val + Ile presented a similar AUC (0.873) with Ile alone, but its sensitivity (83.7%) slightly drops. Although Ile + Leu exhibits a slightly higher AUC (0.884) compared with Ile alone, its specificity (74%) slightly decreases. In addition, the Val + Ile + Leu (AUC = 0.884, sensitivity = 87.8%, specificity = 74%) exhibits a basically same predictive efficiency with Ile + Leu (**Figure 2B** and **Table 5**). Because the combinations do not improve the predictive value of Ile for GDM, Ile is considered to be the most valuable BCAA in the diagnosis of GDM. Furthermore, advanced maternal age (≥ 35) and overweight (BMI ≥ 24) are combined with Ile to predict GDM due to their key roles in the onset of GDM [19-21]. Encouragingly, the combinations of age (≥ 35) or BMI (≥ 24) both improve the predictive value of Ile. The predictive value of the Ile + age (≥ 35) + BMI (≥ 24) (AUC = 0.902, sensitivity = 93.9%, specificity = 80%) is further improved compared with Ile + age (≥ 35) or BMI (≥ 24). However, the Val + Ile + Leu + age (≥ 35) + BMI (≥ 24) fail to show a better predictive value for GDM than Ile + age (≥ 35) + BMI (≥ 24) (**Figure 2C** and **Table 5**). Therefore, Ile + age (≥ 35) + BMI (≥ 24) present a promising application potential in the clinical prediction of GDM.

**Table 5:** The predictive value of BCAAs in gestational diabetes mellitus (GDM) at the first trimester (8-12 weeks).

Markers	Cutoff	Sensitivity (%)	Specificity (%)	AUC (95% CI)	P value
Val	137.142	75.5	70	0.782 (0.693-0.871)	< 0.001^***^
Ile	7.946	85.7	78	0.872 (0.804-0.941)	< 0.001^***^
Leu	9.929	89.8	36	0.669 (0.564-0.775)	0.004^**^
Val + Ile	0.478	83.7	80	0.873 (0.805-0.942)	< 0.001^***^
Val + Leu	0.552	67.3	78	0.781 (0.692-0.870)	< 0.001^***^
Ile + Leu	0.357	87.8	74	0.884 (0.818-0.950)	< 0.001^***^
Val + Ile + Leu	0.357	87.8	74	0.884 (0.818-0.951)	< 0.001^***^
Ile + Age (≥ 35)	0.414	91.8	80	0.879 (0.811-0.947)	< 0.001^***^
Ile + BMI (≥ 24)	0.441	85.7	80	0.888 (0.826-0.951)	< 0.001^***^
Ile + Age (≥ 35) + BMI (≥ 24)	0.349	93.9	80	0.902 (0.842-0.962)	< 0.001^***^
Val + Ile + Leu+ Age (≥ 35) + BMI (≥ 24)	0.372	93.9	80	0.904 (0.845-0.963)	< 0.001^***^

Val, valine; Ile, isoleucine; Leu, leucine; BMI, body mass index, AUC, area under curve; CI, confidence interval. ^**^P < 0.01, ^***^P < 0.001.

## Discussion

This study demonstrated that the serum levels of Val, Ile, and Leu are significantly higher in pregnant women with GDM than the controls in the first trimester, and are significantly lower in the second trimester than the first trimester in GDM patients. In addition, serum Val, Ile, and Leu in the first trimester are the risk factors of GDM, among which Ile has a certain predictive value for GDM. It is worth noting that Ile combined with age (≥ 35) and BMI (≥ 24) is valuable in the early prediction of GDM.

### Transverse comparisons of BCAAs in GDM patients and controls

BCAAs are positively correlated with insulin resistance and the occurrence and development of T2DM (23, 24), whereas their relations with GDM are still controversial. In this study, the dynamic changes of serum BCAAs were analyzed in pregnant women with GDM and normal controls. Transverse comparisons revealed that the serum levels of Val, Ile, and Leu in the first trimester are significantly higher in patients with GDM than those in the controls. These results are consistent with some previous studies. For examples, a study in Finland by Mokkala et al. (2020) has found that the serum levels of Ile and Leu in the first trimester are significantly higher in pregnant women with GDM than those in healthy pregnant women (14); another study in China by Jiang et al. (2020) has revealed that the serum level of Ile in the first trimester is higher in women with GDM compared with that in healthy pregnant women (15). Different with our results, Lewis et al. (2015) have indicated that there no significant changes on the BCAAs were observed in pregnant women with GDM in the first trimester (16). This contradiction may be attributed to the differences on the race, living and dietary habits, age, sampling time, detection methods, etc. In addition, the metabolic characteristics of BCAAs in GDM women, including the increased Ile are similar in pregnant women with pregestational diabetes mellitus (PGDM) during early pregnancy and also in non-pregnant individuals with T2DM (25). Because the T2DM and GDM have similar pathogenesis in some aspects, the elevation of BCAAs in the first trimester may be involved in the progression of GDM in similar mechanisms with that in T2DM. First, BCAAs can induce insulin resistance in different metabolic tissues in specific ways. In skeletal muscles, the accumulation of BCAAs, including Val-produced 3-hydroxy-isobutyrate promotes fatty acid uptake, induces incomplete oxidation of fatty acids, thereby causing skeletal insulin resistance (26, 27). In liver tissues, the decomposition of branched-chain α-keto acids damages mitochondrial tricarboxylic acid cycle, leading to incomplete oxidation products accumulation, mitochondrial stress, and insulin resistance (26, 27). In adipose tissues, the decreased expression of BCAA catabolic enzymes leads to the accumulation of plasma BCAAs, causing deposition of BCAAs in skeletal muscle and liver tissues (26, 27). Second, a long time of elevated plasma levels of BCAAs leads to the hyperactivation of mTOR signaling, inducing insulin receptor substrate degradation (28, 29). Third, BCAAs facilitate glucose uptake by the liver and skeletal muscle and enhance glycogen synthesis in an insulin-independent manner through phosphatidylinositol 3-kinase or protein kinase C pathways (30, 31). However, the detail action mechanisms of BCAAs in GDM remain unclear, which need to be further studied.

With the progressing of pregnancy in the second trimester, Ile is found to be decreased in patients with GDM compared with the controls, while Val and Leu show no significantly differences. Jansson et al. (2002) have revealed that the transportation of Leu by syncytiotrophoblast microvillous plasma membranes is enhanced in patients with GDM combined with large-for-gestational-age fetuses compared with normal pregnant women (32). Cetin I et al. (2005) have found that the Leu, Ile, and Val levels are increased in the umbilical vein and artery of women with GDM, indicating the alteration of placental amino acid exchange during pregnancy (33). Therefore, we speculate that GDM is likely to exacerbate the transportation of early enriched BCAAs to developing tissues, such as the placenta and fetus in the second trimester (34, 35).

### Longitudinal comparisons of BCAAs in the first and second trimesters

Until now, the comparative study on BCAAs between the first and second trimesters is still limited. Zhao et al. (2019) have found that the levels of Leu/Ile and related metabolites in the GDM group are decreased in the second trimester compared with those in the first trimester, while no significant changes are observed in the control group (20). In this study, longitudinal comparisons showed that the serum levels of Val, Ile, and Leu in patients with GDM are significantly lower in the second trimester than those in the first trimester. Our results are not exactly the same as those of Zhao et al. (2019) and indicate that the serum levels of BCAAs are abnormally decreased with the occurrence of GDM. Generally, most amino acids can flow into the fetus through the placenta (36, 37). There is a high activity of branched-chain aminotransferases in the placental tissues, contributing to the uptake of BCAAs for the sustain of high placental nitrogen demand (38). Notably, a previous study on serum metabolic changes has determined that amino acids are decreased during pregnancy in patients with GDM, indicating an increased utilization of amino acids in placenta and fetus (25). Therefore, the decreased serum BCAAs in women with GDM in the second trimester may be partly attributed to the enhanced transportation of BCAAs by the placenta. Besides, serum Ile and Leu are significantly increased in the second trimester compared with the first trimester in normal controls, however, the relevant mechanisms are still clear.

### Early predictive value of BCAAs in GDM

So far, there are few studies on the predictive value of BCAAs in GDM. Evidence has determined that the serum Ile in the first trimester may predict GDM with a AUC of 0.65-0.725 (14, 15). In our study, the predictive value of BCAAs in the first trimester for GDM was further evaluated. Similar to the findings of Mokkala et al. (2020), ROC analysis shows that although Leu has statistical significance in GDM prediction, but its specificity is low (AUC = 0.669, specificity = 36%). In addition, a certain predictive value for GDM is revealed in Val or Ile alone (AUC > 0.65), along with a better role of Ile. Because various combinations of BCAAs fail to improve the predictive value of Ile in GDM, Ile (AUC = 0.872, sensitivity = 85.7%, specificity = 78%) is considered to be the most valuable marker for the prediction of GDM among BCAAs. On the other hand, the occurrence of GDM can typically be afflicted by confounding factors, and many risk factors of GDM have been identified, such as advanced maternal age, obesity, excessive gestational weight gain, westernized diet, micronutrient deficiency, genetic polymorphism, and history of diseases with insulin resistance (39). Because age and BMI are not only closely associated with the risk of GDM (40–43), but also the easiest indicator to count before pregnancy, we further combined advanced maternal age and overweight with Ile for the early prediction of GDM. Encouragingly, both age (≥ 35) and BMI (≥ 24) improve the predictive value of Ile for GDM. A relatively ideal predictive efficiency is shown in the combination of Ile + age (≥ 35) + BMI (≥ 24), with 93.9% sensitivity and 80% specificity.

## Conclusions

The serum levels of Val, Ile, and Leu are significantly higher in pregnant women with GDM than the controls in the first trimester, while the Ile is significantly lower in those with GDM than the controls in the second trimester. In GDM cases, serum Val, Ile, and Leu are lower in the second trimester than the first trimester. Based on diverse regression models, the Val, Ile, and Leu in the first trimester, and the Ile in the second trimester are determined as the risk factors of GDM. In addition, Ile in the first trimester is an effective predictive marker of GDM, which is better than Val and Leu. Ile + age (≥ 35) + BMI (≥ 24) exhibit a relatively ideal predictive value for GDM. Our findings not only reveal the dynamic changes of BCAAs in early and middle pregnancy, but also firstly combine BCAAs with clinical indicators for the prediction of GDM. Ile combined with age (≥ 35) and BMI (≥ 24) may be promising biomarkers for early detection of GDM. However, the insulin concentration is not enrolled in the analyses of this study, and its relations with BCAAs still need to be explored. This study is also limited by the single-center, sample size, and ethnicity. Our conclusions still need to be verified in prospective studies based on multi-center and large sample size in future.

## Data availability statement

The raw data supporting the conclusions of this article will be made available by the authors, without undue reservation.

## Ethics statement

This study was reviewed and approved by Ethics Committee of Beijing Obstetrics and Gynecology Hospital (2017-KY-015-01). The patients/participants provided their written informed consent to participate in this study.

## Author contributions

Conceptualization, XM and GL. Formal analysis, XW and YZ. Investigation, YZ, YW, WS and CG. Data Curation, XW, WZ, JW and SL. Validation, GL. Visualization, XW. Funding acquisition, XM and GL. Writing - Original Draft, XW. Writing - Review and Editing, YZ, XM and GL. All authors contributed to the article and approved the submitted version.

## Funding

This study was supported by National Natural Science Foundation of China (No. 82171671), The National Key Research and Development Program of China (No. 2016YFC1000304), Beijing Hospitals Authority’ Ascent Plan (No. DFL20191402), Scientific Research Common Program of Beijing Municipal Commission of Education (No. KM202110025007), and Beijing Natural Science Foundation (No. 7214231).

## Conflict of interest

The authors declare that the research was conducted in the absence of any commercial or financial relationships that could be construed as a potential conflict of interest.

## Publisher’s note

All claims expressed in this article are solely those of the authors and do not necessarily represent those of their affiliated organizations, or those of the publisher, the editors and the reviewers. Any product that may be evaluated in this article, or claim that may be made by its manufacturer, is not guaranteed or endorsed by the publisher.

## References

[1] Ben-Haroush A Yogev Y Hod M . Epidemiology of gestational diabetes mellitus and its association with type 2 diabetes. Diabetic Med (2010) 21(2):103–13. doi: 10.1046/j.1464-5491.2003.00985.x 14984444

[2] Chen L Mayo R Chatry A Hu G . Gestational diabetes mellitus: Its epidemiology and implication beyond pregnancy. Curr Epidemiol Rep (2016) 3(1):1–11. doi: 10.1007/s40471-016-0063-y

[3] Seshiah V Das AK Balaji V Joshi SR Parikh MN Gupta S . Gestational diabetes mellitus–guidelines. J Assoc Physicians India. (2006) 54:622–8. 16941793

[4] Hossein-Nezhad A Maghbooli Z Vassigh AR Larijani B . Prevalence of gestational diabetes mellitus and pregnancy outcomes in Iranian women. Taiwan J Obstet Gynecol. (2007) 46(3):236–41. doi: 10.1016/S1028-4559(08)60026-1 17962102

[5] Bellamy L Casas JP Hingorani AD Williams D . Type 2 diabetes mellitus after gestational diabetes: A systematic review and meta-analysis. Lancet (2009) 373(9677):1773–9. doi: 10.1016/S0140-6736(09)60731-5 19465232

[6] Sullivan SD Umans JG Ratner R . Gestational diabetes: Implications for cardiovascular health. Curr Diabetes Rep (2012) 12(1):43–52. doi: 10.1007/s11892-011-0238-3 22037824

[7] Hughes AE Hayes MG Egan AM Patel KA Scholtens DM Lowe LP . All thresholds of maternal hyperglycaemia from the WHO 2013 criteria for gestational diabetes identify women with a higher genetic risk for type 2 diabetes. Wellcome Open Res (2020) 5:175. doi: 10.12688/wellcomeopenres.16097.1 33869792 PMC8030121

[8] Juan J Yang H . Prevalence, prevention, and lifestyle intervention of gestational diabetes mellitus in China. Int J Environ Res Public Health (2020)17(24):9517. doi: 10.3390/ijerph17249517 PMC7766930 33353136

[9] Nie C He T Zhang W Zhang G Ma X . Branched chain amino acids: Beyond nutrition metabolism. Int J Mol Sci (2018)19(4):954. doi: 10.3390/ijms19040954 PMC5979320 29570613

[10] Zheng Y Li Y Qi Q Hruby A Manson JE Willett WC . Cumulative consumption of branched-chain amino acids and incidence of type 2 diabetes. Int J Epidemiol. (2016) 45(5):1482–92. doi: 10.1093/ije/dyw143 PMC5100612 27413102

[11] Wang TJ Larson MG Vasan RS Cheng S Rhee EP McCabe E . Metabolite profiles and the risk of developing diabetes. Nat Med (2011) 17(4):448–53. doi: 10.1038/nm.2307 PMC3126616 21423183

[12] Guasch-Ferre M Hruby A Toledo E Clish CB Martinez-Gonzalez MA Salas-Salvado J . Metabolomics in prediabetes and diabetes: A systematic review and meta-analysis. Diabetes Care (2016) 39(5):833–46. doi: 10.2337/dc15-2251 PMC4839172 27208380

[13] Tobias DK Clish C Mora S Li J Liang L Hu FB . Dietary intakes and circulating concentrations of branched-chain amino acids in relation to incident type 2 diabetes risk among high-risk women with a history of gestational diabetes mellitus. Clin Chem (2018) 64(8):1203–10. doi: 10.1373/clinchem.2017.285841 PMC6434682 29945965

[14] Mokkala K Vahlberg T Pellonpera O Houttu N Koivuniemi E Laitinen K . Distinct metabolic profile in early pregnancy of overweight and obese women developing gestational diabetes. J Nutr (2020) 150(1):31–7. doi: 10.1093/jn/nxz220 31529056

[15] Jiang R Wu S Fang C Wang C Yang Y Liu C . Amino acids levels in early pregnancy predict subsequent gestational diabetes. J Diabetes. (2020) 12(7):503–11. doi: 10.1111/1753-0407.13018 31883199

[16] Bentley-Lewis R Huynh J Xiong G Lee H Wenger J Clish C . Metabolomic profiling in the prediction of gestational diabetes mellitus. Diabetologia (2015) 58(6):1329–32. doi: 10.1007/s00125-015-3553-4 PMC4428592 25748329

[17] Zhao L Wang M Li J Bi Y Li M Yang J . Association of circulating branched-chain amino acids with gestational diabetes mellitus: A meta-analysis. Int J Endocrinol Metab (2019) 17(3):e85413. doi: 10.5812/ijem.85413 31497040 PMC6679587

[18] Park S Park JY Lee JH Kim SH . Plasma levels of lysine, tyrosine, and valine during pregnancy are independent risk factors of insulin resistance and gestational diabetes. Metab Syndr Relat Disord (2015) 13(2):64–70. doi: 10.1089/met.2014.0113 25419905

[19] Rahimi N Razi F Nasli-Esfahani E Qorbani M Shirzad N Larijani B . Amino acid profiling in the gestational diabetes mellitus. J Diabetes Metab Disord (2017) 16:13. doi: 10.1186/s40200-016-0283-1 28367428 PMC5374565

[20] Zhao H Li H Chung ACK Xiang L Li X Zheng Y . Large-Scale longitudinal metabolomics study reveals different trimester-specific alterations of metabolites in relation to gestational diabetes mellitus. J Proteome Res (2019) 18(1):292–300. doi: 10.1021/acs.jproteome.8b00602 30488697

[21] Mack LR Tomich PG . Gestational diabetes: Diagnosis, classification, and clinical care. Obstet Gynecol Clin North Am (2017) 44(2):207–17. doi: 10.1016/j.ogc.2017.02.002 28499531

[22] Chinese Ministry of Health . Guidelines for prevention and control of overweight and obesity in Chinese adults. People's Med Publishing House (2006) 17, 1.

[23] Menni C Fauman E Erte I Perry JR Kastenmuller G Shin SY . Biomarkers for type 2 diabetes and impaired fasting glucose using a nontargeted metabolomics approach. Diabetes (2013) 62(12):4270–6. doi: 10.2337/db13-0570 PMC3837024 23884885

[24] Palmer ND Stevens RD Antinozzi PA Anderson A Bergman RN Wagenknecht LE . Metabolomic profile associated with insulin resistance and conversion to diabetes in the insulin resistance atherosclerosis study. J Clin Endocrinol Metab (2015) 100(3):E463–8. doi: 10.1210/jc.2014-2357 PMC4333040 25423564

[25] Walejko JM Chelliah A Keller-Wood M Wasserfall C Atkinson M Gregg A . Diabetes leads to alterations in normal metabolic transitions of pregnancy as revealed by time-course metabolomics. Metabolites (2020) 10(9):350. doi: 10.3390/metabo10090350 PMC7570364 32867274

[26] Newgard CB . Interplay between lipids and branched-chain amino acids in development of insulin resistance. Cell Metab (2012) 15(5):606–14. doi: 10.1016/j.cmet.2012.01.024 PMC3695706 22560213

[27] Shou J Chen PJ Xiao WH . The effects of BCAAs on insulin resistance in athletes. J Nutr Sci Vitaminol (Tokyo). (2019) 65(5):383–9. doi: 10.3177/jnsv.65.383 31666474

[28] Xie J Herbert TP . The role of mammalian target of rapamycin (mTOR) in the regulation of pancreatic beta-cell mass: implications in the development of type-2 diabetes. Cell Mol Life Sci (2012) 69(8):1289–304. doi: 10.1007/s00018-011-0874-4 PMC11114779 22068611

[29] Krebs M Krssak M Bernroider E Anderwald C Brehm A Meyerspeer M . Mechanism of amino acid-induced skeletal muscle insulin resistance in humans. Diabetes (2002) 51(3):599–605. doi: 10.2337/diabetes.51.3.599 11872656

[30] Nishitani S Matsumura T Fujitani S Sonaka I Miura Y Yagasaki K . Leucine promotes glucose uptake in skeletal muscles of rats. Biochem Biophys Res Commun (2002) 299(5):693–6. doi: 10.1016/S0006-291X(02)02717-1 12470633

[31] Monirujjaman M Ferdouse A . Metabolic and physiological roles of branched-chain amino acids. Adv Mol Biol (2014) 2014:364976. doi: 10.1155/2014/364976

[32] Jansson T Ekstrand Y Bjorn C Wennergren M Powell TL . Alterations in the activity of placental amino acid transporters in pregnancies complicated by diabetes. Diabetes (2002) 51(7):2214–9. doi: 10.2337/diabetes.51.7.2214 12086952

[33] Cetin I de Santis MS Taricco E Radaelli T Teng C Ronzoni S . Maternal and fetal amino acid concentrations in normal pregnancies and in pregnancies with gestational diabetes mellitus. Am J Obstet Gynecol. (2005) 192(2):610–7. doi: 10.1016/j.ajog.2004.08.011 15696011

[34] Allman BR Diaz EC Andres A Borsheim E . Divergent changes in serum branched-chain amino acid concentrations and estimates of insulin resistance throughout gestation in healthy women. J Nutr (2020) 150(7):1757–64. doi: 10.1093/jn/nxaa096 PMC7330471 32275314

[35] Kalkhoff RK Kandaraki E Morrow PG Mitchell TH Kelber S Borkowf HI . Relationship between neonatal birth weight and maternal plasma amino acid profiles in lean and obese nondiabetic women and in type I diabetic pregnant women. Metabolism (1988) 37(3):234–9. doi: 10.1016/0026-0495(88)90101-1 3343932

[36] Battaglia FC Meschia G . Principal substrates of fetal metabolism. Physiol Rev (1978) 58(2):499–527. doi: 10.1152/physrev.1978.58.2.499 417347

[37] Lemons JA Adcock EW 3rd Jones MD Jr. Naughton MA Meschia G Battaglia FC . Umbilical uptake of amino acids in the unstressed fetal lamb. J Clin Invest. (1976) 58(6):1428–34. doi: 10.1172/JCI108598 PMC333314 1033209

[38] Battaglia FC Regnault TR . Placental transport and metabolism of amino acids. Placenta (2001) 22(2-3):145–61. doi: 10.1053/plac.2000.0612 11170819

[39] Plows JF Stanley JL Baker PN Reynolds CM Vickers MH . The pathophysiology of gestational diabetes mellitus. Int J Mol Sci (2018) 19(11):3342. doi: 10.3390/ijms19113342 PMC6274679 30373146

[40] Giannakou K Evangelou E Yiallouros P Christophi CA Middleton N Papatheodorou E . Risk factors for gestational diabetes: An umbrella review of meta-analyses of observational studies. PloS One (2019) 14(4):e0215372. doi: 10.1371/journal.pone.0215372 31002708 PMC6474596

[41] Lean SC Derricott H Jones RL Heazell AEP . Advanced maternal age and adverse pregnancy outcomes: A systematic review and meta-analysis. PloS One (2017) 12(10):e0186287. doi: 10.1371/journal.pone.0186287 29040334 PMC5645107

[42] Lee KW Ching SM Ramachandran V Yee A Hoo FK Chia YC . Prevalence and risk factors of gestational diabetes mellitus in Asia: A systematic review and meta-analysis. BMC Pregnancy Childbirth. (2018) 18(1):494. doi: 10.1186/s12884-018-2131-4 30547769 PMC6295048

[43] Lowe WL Jr. Bain JR Nodzenski M Reisetter AC Muehlbauer MJ Stevens RD . Erratum. maternal BMI and glycemia impact the fetal metabolome. Diabetes Care (2017) 40:902–10. doi: 10.2337/dc16-2452 PMC5481987 28637888

